# Disease-specific divergence of inflammatory and metabolic biomarkers in neurocritical neuromuscular disorders

**DOI:** 10.3389/fneur.2026.1820825

**Published:** 2026-05-13

**Authors:** Sezgin Kehaya, Erdi Şensöz

**Affiliations:** Department of Neurology, Faculty of Medicine, Trakya University, Edirne, Türkiye

**Keywords:** Guillain–Barré syndrome, lactate dehydrogenase, mechanical ventilation, myasthenia gravis, neutrophil-to-lymphocyte ratio, prognosis, systemic immune–inflammation index

## Abstract

**Background:**

Myasthenia gravis (MG) and Guillain–Barré syndrome (GBS) are immune mediated neuromuscular disorders that may require intensive immunotherapy and respiratory support. Although inflammatory biomarkers have been explored in both conditions, their diagnosis specific prognostic value remains unclear. We aimed to compare hemogram-derived inflammatory indices and metabolic injury-related biomarkers in hospitalized MG and GBS patients and to evaluate their associations with disease severity and clinical outcomes.

**Methods:**

This retrospective cohort study included 162 patients (88 MG, 74 GBS) treated with intravenous immunoglobulin and/or plasma exchange. Hemogram-derived indices (neutrophil-to-lymphocyte ratio [NLR], systemic immune–inflammation index [SII]), classical inflammatory markers, and metabolic biomarkers including lactate dehydrogenase (LDH) and the LDH to albumin ratio (LAR) were analyzed in relation to neurological severity, length of hospital stay (LOS), and mechanical ventilation (MV). Receiver operating characteristic analyses and diagnosis-specific multivariable logistic regression models were performed.

**Results:**

Mechanical ventilation occurred in 10.5% of patients and was strongly associated with baseline neurological severity in both disorders (*p* < 0.001). In MG, hemogram-derived indices (particularly NLR and SII) demonstrated good discriminatory performance for severe disease and were associated with adverse outcomes. LDH-based parameters, particularly LAR, which may reflect metabolic stress, were associated with disease severity and respiratory involvement in MG. In GBS, outcomes were predominantly determined by neurological severity measures, whereas inflammatory indices showed limited and inconsistent prognostic value. Post-treatment transaminase elevations were modestly associated with more severe disease in GBS. In multivariable models, baseline clinical severity remained the most consistent determinant of mechanical ventilation across both conditions.

**Conclusions:**

Biomarker utility differs between MG and GBS. In MG, inflammatory indices and LDH-based parameters, particularly the LAR, were associated with disease severity and may support risk stratification, including identification of patients at risk for respiratory deterioration. These findings are exploratory and require prospective validation. In GBS, outcomes remain primarily determined by neurological severity.

## 1 Introduction

Guillain–Barré syndrome (GBS) and myasthenia gravis (MG) are distinct autoimmune neuromuscular disorders characterized by immune-mediated dysfunction of the peripheral nervous system and neuromuscular junction, respectively (1, 2). GBS typically presents as an acute inflammatory polyradiculoneuropathy with rapidly progressive symmetrical weakness and potential respiratory failure, whereas MG is a chronic disorder causing fluctuating muscle weakness due to impaired neuromuscular transmission (1, 2). Despite their well-established pathophysiological differences, both conditions may require hospitalization, intensive immunotherapy, and close monitoring due to the risk of respiratory failure.

Systemic inflammation plays an important role in the pathophysiology of both conditions. Hemogram-derived inflammatory indices such as the neutrophil-to-lymphocyte ratio (NLR), platelet-to-lymphocyte ratio (PLR), and systemic immune–inflammation index (SII) have emerged as accessible and cost-effective markers associated with disease activity, respiratory failure, and outcomes in neuromuscular autoimmune disorders (3–8). However, the majority of previous studies have evaluated these biomarkers within single-disease cohorts, limiting the understanding of whether their prognostic performance is disease-specific or generalizable across neuroimmune conditions.

In addition to hemogram-based indices, biochemical markers reflecting tissue injury may provide complementary prognostic information. Elevated alanine aminotransferase (ALT) and aspartate aminotransferase (AST) levels have been associated with worse outcomes in GBS, possibly reflecting systemic stress or secondary organ involvement (9). Lactate dehydrogenase (LDH), released during cellular injury, has also been linked to inflammatory activation and adverse outcomes in systemic conditions (10, 11). The LDH/albumin ratio has recently been proposed as a composite marker reflecting both tissue injury and physiological reserve; however, its role in neuromuscular autoimmune disorders, particularly in MG, remains largely unexplored. Importantly, MG and GBS differ not only in pathophysiology but also in the relative contribution of systemic inflammation vs. structural neurological damage to clinical outcomes, raising the question of whether commonly used biomarkers provide comparable prognostic value across these conditions.

Therefore, this study aimed to comparatively evaluate the association of systemic inflammatory and injury-related biomarkers with disease severity, mechanical ventilation requirement, and prognosis in hospitalized patients with MG and GBS. Rather than directly comparing biological mechanisms, this study specifically investigates whether routinely available biomarkers demonstrate consistent or disease-specific prognostic behavior under comparable neurocritical conditions requiring intravenous immunoglobulin and/or plasma exchange. By focusing on a hospitalized cohort requiring intensive immunotherapy, this study reflects a more severe disease spectrum, and therefore findings should be interpreted within the context of neurocritical care rather than general outpatient populations.

## 2 Materials and methods

After obtaining approval from the Non Interventional Scientific Research Ethics Committee of Trakya University Faculty of Medicine (Approval No: TÜTF-GOBEAK 2024/327, Decision: 13/13, Date: 19.08.2024), hospitalized patients between August 2015 and August 2023 in the Neurology Department were retrospectively reviewed. Written informed consent for using clinical and laboratory data for research was obtained from all patients, hospitalized at Trakya University Medical Faculty and no additional consent was required. All procedures complied with the Declaration of Helsinki and local ethical regulations.

MG diagnosis was established based on clinical criteria supported by serological tests for anti-acetylcholine receptor (AChR) antibodies, anti-MuSK antibodies, and electrodiagnostic findings when appropriate (2). Patients with bulbar or generalized weakness were included to the study. These inclusion criteria were chosen to focus on patients with clinically significant disease requiring hospitalization and immunomodulatory therapy, rather than representing the full spectrum of MG severity. GBS diagnosis was made according to clinical presentation and electrophysiological criteria, with subtyping into acute inflammatory demyelinating polyneuropathy (AIDP), acute motor axonal neuropathy (AMAN), acute motor-sensory axonal neuropathy (AMSAN) and Miller Fisher syndrome (MFS) based on nerve conduction studies (1). Electrophysiological classification was performed according to standard nerve conduction criteria used in clinical practice. Patients with incomplete clinical data, receiving treatment for active other autoimmune disease or malignancies, patients with active infection at admission were excluded to minimize confounding effects on systemic inflammatory biomarkers (7, 8). These exclusion criteria were applied to reduce potential confounding factors affecting systemic inflammatory and metabolic biomarkers. Pregnant women or patients under 18 years of age were excluded, too.

Demographic data, comorbidities such as hypertension and diabetes, thymectomy status for MG, syndrome of inappropriate antidiuretic hormone (SIADH) secretion and disease specific severity scores were collected. For MG patients, antibody status (anti-AChR, anti-MuSK, seronegative, and undetermined) was also recorded and summarized to reflect disease heterogeneity. Disease severity and functional status in MG were evaluated using standardized clinical tools: Quantitative Myasthenia Gravis (QMG) score at admission, discharge, 6 months, and 1st year: a 13-item physician administered scale measuring muscle strength, with higher scores indicating more severe weakness (12, 13). Myasthenia Gravis Foundation of America (MGFA) clinical classification: Categorizes patients into classes I to V according to the extent and severity of muscle involvement (13, 14). For GBS patients, disease severity and prognosis were assessed using: Modified Erasmus GBS Outcome Score (mEGOS) at admission and during follow up (15). Medical Research Council (MRC) sum score at admission, discharge, 6 months, and 1st year to assess muscle strength recovery, like previous studies (16, 17). GBS subtype-specific characteristics were considered in the interpretation of outcomes, given the known differences between demyelinating and axonal variants. Mechanical ventilation (MV) requirement and length of hospital stay (LOS) were also recorded as indicators of disease severity. For prognostic analyses, outcome measures were evaluated within each disease group using validated disease-specific scales and were not intended for direct cross-disease comparison. For prognostic analyses, poor outcome was defined as MGFA class ≥4 in MG and GBS Disability Score ≥3 in GBS. Disease severity was defined as QMG score ≥ median in MG and the most severe mEGOS ≥ median in GBS (14, 15). These definitions were selected to reflect clinically meaningful thresholds within each disease rather than to establish direct comparability between MG and GBS.

Analyzed laboratory tests were complete blood count (CBC): including total white blood cell (WBC) count and differential (neutrophils, lymphocytes, monocytes), hemoglobin, hematocrit, mean corpuscular volume (MCV), and platelet count. Biochemical tests included serum levels of liver enzymes (ALT, AST, gamma glutamyl transferase [GGT]), LDH, total protein, albumin, urea, creatinine, and electrolytes (sodium, potassium, chloride). Autoantibody profiles were noted for MG patients (anti-AChR, anti-MuSK, seronegative status). Cerebrospinal fluid (CSF) cell count and protein levels were noted for GBS patients. From these values, systemic inflammatory indices were derived to assess the inflammatory state: neutrophil-to-lymphocyte ratio (NLR), platelet-to-lymphocyte ratio (PLR), monocyte-to-lymphocyte ratio (MLR), lymphocyte-to-monocyte ratio (LMR), systemic immune–inflammation index (SII), and systemic inflammatory response index (SIRI). Calculations were performed according to previous studies (3–8). These indices were selected based on prior evidence supporting their association with disease severity and outcomes in neuromuscular and autoimmune disorders (4, 5). The LDH/albumin ratio (LAR) was calculated as a composite biomarker reflecting tissue injury and physiological reserve, integrating metabolic and inflammatory components, as previously described (10, 11). Transaminase levels (ALT and AST) were evaluated as part of systemic metabolic response; however, their interpretation considered potential influences of disease severity and treatment-related effects. Given the retrospective design, not all laboratory parameters (e.g., CK levels) were consistently available for all patients. Treatment regimens included intravenous immunoglobulin (IVIG), plasma exchange (PE), and combination therapy (PE + IVIG). Treatment modality was recorded but interpreted cautiously due to its strong association with baseline disease severity.

Statistical analyses were performed using SPSS version 22.0. Continuous variables were expressed as mean ± SD or median (IQR), as appropriate. Group comparisons were performed using independent-samples *t*-test or Mann–Whitney *U*–test for continuous variables and chi-square or Fisher's exact test for categorical variables. Correlations between biomarkers and clinical severity scores were assessed using Pearson or Spearman correlation analyses according to data distribution. Receiver operating characteristic (ROC) curve analyses were conducted to evaluate the discriminatory performance of biomarkers, and area under the curve (AUC) values were compared using the DeLong test. Given the limited number of mechanical ventilation (MV) events, multivariable regression analyses were constructed using parsimonious models to reduce the risk of overfitting. Multivariable analyses were performed separately within MG and GBS groups to account for differences in disease mechanisms, severity scales, and clinical trajectories. Results of multivariable analyses were interpreted cautiously and considered exploratory rather than definitive. No formal correction for multiple comparisons was applied; therefore, findings should be interpreted as hypothesis-generating. A two-tailed *p*-value < 0.05 was considered statistically significant.

No generative artificial intelligence was used in the study.

## 3 Results

### 3.1 Baseline demographic, clinical, and laboratory characteristics

A total of 162 patients were included (88 MG, 74 GBS), with mechanical ventilation (MV) occurring in 17 patients (10.5%). Baseline demographic, clinical, and laboratory characteristics are summarized in Table 1 (detailed in Supplementary Table 1). The cohort represents hospitalized patients requiring immunomodulatory therapy and therefore reflects a relatively severe clinical spectrum.

**Table 1:** Baseline demographic, clinical, and selected laboratory characteristics of patients with MG and GBS.

Variable	MG *n* = 88	GBS *n* = 74	*p*-value
Demographic and clinical characteristics			
Male sex, %	54.5	56.8	0.451
Age, years (mean ± SD)	62.44 ± 16.4	57.26 ± 16.5	**0.048**
Hypertension, %	50.0	44.6	0.530
Diabetes mellitus, %	23.9	18.9	0.566
Mechanical ventilation, %	9.1	12.2	0.610
Length of hospital stay, days (mean ± SD)	11.24 ± 15.2	14.20 ± 8.7	0.137
SIADH, % (*n*)	3.4 (3)	10.8 (8)	0.114
Treatment modality, %			
IVIG	89.8	77.0	**0.041**
Plasma exchange (PE)	4.5	16.2	**–**
IVIG + PE	5.7	6.8	**–**
Hematological parameters			
Hemoglobin, g/dl	12.8 ± 2.0	13.5 ± 1.9	**0.019**
Platelet count ( × 10^3^/μl)	251.5 ± 74	280.4 ± 101	0.038
Inflammatory indices			
NLR	6.3 ± 11.1	4.5 ± 3.8	0.119
SII	1410 ± 1915	1341 ± 1490	0.800
ESR, mm/h	21.9 ± 18.0	29.4 ± 21.0	**0.020**
CRP, mg/dl	1.29 ± 3.8	1.68 ± 2.6	0.470
Biochemical parameters			
Total protein, g/dl	6.96 ± 0.8	7.24 ± 0.8	**0.030**
Albumin, g/dl	3.8 ± 0.5	3.8 ± 0.5	0.618
Admission sodium, mmol/L	139 ± 3.6	137 ± 4.0	**< 0.001**
Admission chloride, mmol/L	104 ± 4	102 ± 5	**0.010**
GGT, U/L	29 ± 21	50 ± 70	0.023
LDH (admission), U/L	289 ± 156	321 ± 130	0.538
LDH (1st week), U/L	277 ± 190	258 ± 107	0.524
LDH/albumin (admission)	80.18 ± 50.2	84.9 ± 35.8	0.538
LDH/albumin (1st week)	76.76 ± 59.2	70.6 ± 38.8	0.528
ALT (admission), U/L	19.68 ± 10.9	29.04 ± 23.7	**0.001**
AST (admission), U/L	23.11 ± 8.5	31.88 ± 14.6	**< 0.001**
ALT (after treatment), U/L	32.09 ± 33.8	56.24 ± 68.0	**0.004**

Values are presented as mean ± standard deviation or percentage, as appropriate. Variables with *p* < 0.05 are highlighted to emphasize clinically relevant differences. Comparisons between groups were performed using the independent-samples *t*-test or Mann–Whitney U-test for continuous variables, and the χ^2^ test or Fisher's exact test for categorical variables. Treatment modality distribution reflects baseline disease severity and should not be interpreted as an independent determinant. The study population consists of hospitalized patients requiring immunomodulatory therapy and therefore represents a relatively severe clinical spectrum. Inflammatory and biochemical parameters represent systemic measurements and should be interpreted in the context of disease severity and clinical status. SIADH, syndrome of inappropriate antidiuretic hormone secretion; ESR, erythrocyte sedimentation rate; CRP, C-reactive protein; GGT, gamma-glutamyl transferase. Bold values indicate statistically significant differences (*p* < 0.05).

### 3.2 Subtype, severity and treatment properties of patients

Among MG patients, 63.6% were anti-AChR positive, 5.7% anti-MuSK positive, and 15.9% seronegative, while receptor status was undetermined in 14.8%. Antibody status was summarized to reflect disease heterogeneity and was considered in the interpretation of clinical outcomes. Thymectomy had been performed in 17% of cases. According to MGFA classification, most patients were in classes 2a−2b, with smaller proportions in advanced classes. Treatment allocation was strongly severity-driven (*p* < 0.001), with IVIG predominantly administered to milder cases and PE or PE+IVIG used more frequently in advanced MGFA classes. Treatment modality primarily reflected baseline disease severity rather than an independent therapeutic effect. QMG scores differed significantly across treatment groups. Patients treated with PE or PE + IVIG had higher baseline and discharge QMG scores compared with the IVIG group, reflecting greater disease severity (all *p* < 0.01). These differences persisted at 6-month follow-up but were no longer significant at 1 year. Improvement in QMG during hospitalization was greater in patients receiving PE-based therapies (*p* < 0.001).

In GBS patients, subtypes included AIDP (47.3%), AMSAN (32.4%), AMAN (12.2%), and Miller Fisher syndrome (5.1%). The relatively high proportion of axonal variants (AMSAN) in this cohort may reflect regional or referral characteristics and should be considered when interpreting generalizability. Admission and peak mEGOS scores differed among subtypes, with axonal variants demonstrating higher severity (*p* < 0.05). This pattern is consistent with the known association between axonal subtypes and more severe clinical presentation. Treatment selection was similarly associated with baseline severity, with higher mEGOS scores observed in patients receiving PE or combination therapy (*p* < 0.05). Similar to MG, treatment allocation in GBS reflects disease severity, reinforcing the presence of confounding by indication. MRC sum scores varied according to treatment group and subtype. Patients receiving combined therapy had lower baseline and discharge MRC scores, consistent with more severe disease (*p* < 0.01). Differences diminished over long-term follow-up. Axonal subtypes were associated with lower early MRC scores compared with demyelinating forms, although long-term functional recovery did not significantly differ. Outcome patterns across subtypes were considered in interpretation, although subgroup analyses were limited by sample size. Formal subgroup outcome analyses (e.g., LOS or MV according to GBS subtype) were not performed due to limited statistical power within each subtype group and should therefore be interpreted cautiously.

### 3.3 Outcome measures

#### 3.3.1 Primary outcome: mechanical ventilation (MV)

In MG, MV was strongly associated with advanced MGFA class (classes 4–5; *p* < 0.001, ρ = 0.554) and treatment selection (*p* < 0.001, ρ = 0.684). MV also correlated with admission QMG (*p* < 0.001, ρ = 0.464), discharge QMG (*p* < 0.001, ρ = 0.465), 6-month QMG (*p* = 0.001, ρ = 0.380), and length of hospital stay (*p* < 0.001, ρ = 0.455). Mechanical ventilation in MG was closely linked to baseline clinical severity. Among inflammatory biomarkers, MV demonstrated moderate correlations with hemogram-derived indices (NLR ρ = 0.426, *p* < 0.001; SII ρ = 0.412, *p* < 0.001; SIRI ρ = 0.327, *p* = 0.002; PLR ρ = 0.367, *p* < 0.001) and metabolic injury markers (admission LDH ρ = 0.368, *p* = 0.003; first-week LDH ρ = 0.497, *p* < 0.001; admission LDH/albumin ρ = 0.391, *p* = 0.002; first-week LDH/albumin ρ = 0.499, *p* < 0.001). Systemic inflammatory and metabolic markers paralleled disease severity in MG rather than independently determining outcomes. Weak but significant associations were observed for urea (*r* = 0.216, *p* = 0.044), total protein (*r* = −0.301, *p* = 0.004), albumin (*r* = −0.256, *p* = 0.016), first-week ALT (*r* = 0.230, *p* = 0.031), and first-week AST (*r* = 0.416, *p* < 0.001). Baseline CRP correlated with MV (ρ ≈ 0.369, *p* ≈ 0.0004), whereas ESR showed only borderline significance (ρ ≈ 0.209, *p* ≈ 0.051).

In GBS, mechanical ventilation (MV) was strongly associated with neurological disability, particularly GBS Disability Score (GBS-DS; *p* < 0.001, ρ = 0.593), and moderately associated with the most severe mEGOS (*p* = 0.006, ρ = 0.317), length of hospital stay (*p* < 0.001, ρ = 0.433), discharge MRC score (inverse correlation; *p* = 0.001, ρ = −0.364), treatment selection (*p* = 0.005, ρ = 0.320), and WBC count (*p* = 0.004, ρ = 0.331). Mechanical ventilation in GBS was predominantly driven by neurological severity rather than systemic inflammatory burden. Post-treatment liver enzymes also showed moderate correlations with MV (ALT ρ = 0.301, *p* = 0.009; AST ρ = 0.274, *p* = 0.018). These associations may reflect treatment intensity and overall disease severity rather than direct biological effects. In contrast, baseline CRP (*p* ≈ 0.56) and ESR (*p* ≈ 0.60) were not associated with MV. Hemogram-derived indices (NLR, PLR, SII, SIRI) demonstrated weak or non-significant relationships with MV.

Overall, MV in MG was associated with both clinical severity and systemic inflammatory burden, whereas in GBS it was predominantly driven by neurological impairment. In multivariable models (Table 2), baseline clinical severity remained the most consistent determinant of MV, while inflammatory biomarkers did not retain independent significance.

**Table 2:** Multivariable logistic regression analysis for mechanical ventilation requirement.

Myasthenia gravis (*n* = 88, MV events = 8)			
Variable	Odds ratio (OR)	95% CI	*p*–value
Admission QMG (per 1 point)	1.43	1.18–1.75	**< 0.001**
CRP (per 1 mg/dl)	1.16	0.99–1.35	0.063
Age (per 1 year)	1.02	0.94–1.10	0.641
Guillain–Barré syndrome (*n* = 74, MV events = 9)			
CRP (per 1 mg/dl)	0.79	0.42–1.49	0.460
ESR (per 1 mm/h)	0.97	0.91–1.03	0.301
Severe mEGOS (per 1 point)	1.27	0.97–1.65	0.081

Separate diagnosis-specific models were constructed due to differing clinical severity scales. Models were intentionally parsimonious due to the limited number of mechanical ventilation events, and results should be interpreted as exploratory. Baseline clinical severity measures were prioritized in model construction. ORs represent per-unit increases in continuous predictors. In MG, admission QMG was the only independent predictor of mechanical ventilation, whereas inflammatory biomarkers did not retain independent significance after adjustment. In GBS, none of the evaluated variables reached independent significance, indicating that mechanical ventilation risk is predominantly driven by neurological severity rather than systemic inflammatory markers. These findings should be interpreted in the context of limited sample size and require validation in larger prospective cohorts. Given the limited number of events, results should be interpreted as exploratory. Bold values indicate statistically significant differences (*p* < 0.05).

#### 3.3.2 Treatment-stratified predictive performance (ROC/AUC) for MV

CRP demonstrated moderate discrimination for MV in the overall cohort, whereas ESR showed limited predictive value (Table 3). CRP consistently outperformed ESR across treatment strata, although performance varied by subgroup and was limited in patients receiving combined therapy. These findings further support the dominant role of baseline disease severity and the influence of treatment-related confounding.

**Table 3:** Effect of CRP and ESR on MV need.

Baseline inflammatory markers according to mechanical ventilation requirement				
Variable		MV required (*n* = 17)	No MV (*n* = 145)	*p*-value
CRP, mg/dl, median (IQR)		0.78 (0.39–3.12)	0.35 (0.31–1.09)	**0.0016**
ESR, mm/h, median (IQR)		23 (12–42)	18 (10–33)	0.255
Discriminatory performance of CRP and ESR for mechanical ventilation according to treatment strategy				
Treatment group	*n*	MV events	CRP AUC	ESR AUC
IVIG	136	6	0.651	0.538
Plasma exchange	16	4	0.833	0.531
IVIG + PE	10	7	0.476	0.333

Continuous variables are presented as median (interquartile range). Comparisons were per-formed using the Mann-Whitney U-test.

A: Higher CRP levels were observed in patients requiring mechanical ventilation; however, this association should be interpreted in the context of overall disease severity. ESR did not demonstrate a significant association with mechanical ventilation. B: AUC values represent the discriminatory performance of biomarkers for mechanical ventilation. The higher AUC observed in the plasma exchange group should be interpreted cautiously due to small sample size and potential confounding by indication. In the combined treatment group, low AUC values likely reflect the concentration of more severe cases receiving intensified therapy. Overall, biomarker-based discrimination remained modest and secondary to clinical severity in determining mechanical ventilation risk. These findings should be interpreted as exploratory and hypothesis-generating. Bold values indicate statistically significant differences (*p* < 0.05).

### 3.4 Secondary outcomes: LOS, disease severity, prognosis and inflammatory markers

#### 3.4.1 Length of hospital stay

In MG, length of hospital stay (LOS) was strongly associated with mechanical ventilation (*r* = 0.703, *p* < 0.001), treatment selection (*r* = 0.703, *p* < 0.001), MGFA class (*r* = 0.601, *p* < 0.001), and admission QMG score (*r* = 0.642, *p* < 0.001), with similar correlations observed for discharge and 6-month QMG scores (both *p* < 0.001). Prolonged hospitalization in MG was primarily driven by baseline disease severity and clinical course. Among laboratory parameters, LOS showed moderate correlations with hemogram-derived inflammatory indices, particularly NLR and SII, and a weaker association with PLR. Inflammatory markers accompanied disease burden but do not independently determine length of stay. ESR demonstrated a weak correlation with LOS, whereas CRP was not significantly associated. First-week LDH/albumin ratio and creatinine showed weak but significant correlations, suggesting a contribution of metabolic and renal factors to prolonged hospitalization. These metabolic associations should be interpreted cautiously and may reflect overall physiological stress rather than specific disease mechanisms.

Additional analyses were performed to explore the relationship between disease-specific biological features and clinical outcomes. In MG, receptor status showed a weak but significant association with mechanical ventilation requirement (ρ ≈ 0.23, *p* ≈ 0.03), whereas no significant association was observed with length of hospital stay. Thymectomy status was inversely associated with receptor positivity (ρ ≈ −0.33, *p* = 0.002), but was not associated with mechanical ventilation or hospitalization duration.

In GBS, LOS was primarily associated with neurological severity measures, including MV (*r* = 0.561, *p* < 0.001), GBS Disability Score (*r* = 0.552, *p* < 0.001), discharge MRC score (inverse; *r* = −0.500, *p* < 0.001), and the most severe mEGOS (*r* = 0.435, *p* < 0.001). Hospitalization duration in GBS was predominantly determined by neurological impairment. Moderate correlations were also observed for admission mEGOS (*r* = 0.307, *p* = 0.008) and treatment selection (*r* = 0.351, *p* = 0.002). Post-treatment liver enzymes demonstrated notable associations with LOS (ALT *r* = 0.480; AST *r* = 0.413; both *p* < 0.001). These associations likely reflect treatment intensity and disease severity rather than direct biological effects. CRP and ESR were not significantly related. Hemogram-derived indices (NLR *r* = 0.444; SII *r* = 0.446; PLR *r* = 0.377) showed moderate correlations, but these associations were less pronounced compared with neurological severity measures. No significant associations were observed between electrophysiological subtype or CSF parameters and MV or LOS.

Overall, inflammatory biomarkers demonstrated secondary associations compared with neurological severity indicators. These findings suggest that disease-specific biological classifications had limited additional predictive value for short-term clinical outcomes beyond baseline clinical severity measures.

#### 3.4.2 Disease severity and prognosis

In MG, disease severity demonstrated consistent associations with systemic inflammatory burden. Higher MGFA classes correlated moderately with NLR and SII, as well as with the LDH/albumin ratio (ρ ≈ 0.372, *p* = 0.003). Systemic inflammatory and metabolic markers paralleled clinical severity in MG. Admission QMG scores showed moderate correlations with NLR, SII, and SIRI and with LDH-based parameters (ρ ≈ 0.406, *p* = 0.001). These associations support the relevance of systemic biomarkers in reflecting disease burden in MG. Baseline CRP demonstrated a modest association with MGFA but did not correlate with QMG scores, whereas ESR showed weak or inconsistent associations.

In GBS, disease severity and prognosis were predominantly determined by neurological measures. When prognosis was analyzed dichotomously (GBS-DS ≥3), hemogram-derived indices showed weak associations, including NLR, while SII and PLR demonstrated similar low-to-moderate correlations. Systemic inflammatory markers contributed minimally to prognostic assessment in GBS. Post-treatment ALT and AST levels were also weakly associated with poor outcome (*r* ≈ 0.208, *p* ≈ 0.039). These associations may reflect treatment-related effects and overall disease severity rather than independent biological mechanisms. In contrast, CRP and ESR were not significantly related to disease severity or binary prognosis.

Receiver operating characteristic (ROC) analyses further supported these differences. In MG, prognostic discrimination for poor outcome (MGFA ≥4) was excellent for NLR (AUC = 0.95) and SII (AUC = 0.93), followed by CRP (AUC = 0.84), whereas ESR showed limited performance (AUC = 0.66). These high AUC values should be interpreted cautiously and may reflect sample size and cohort characteristics. In GBS, discrimination for poor prognosis (GBS-DS ≥3) was modest for all evaluated biomarkers (NLR AUC = 0.60; SII = 0.59; CRP = 0.52; ESR = 0.53). These results reinforce the limited prognostic utility of systemic biomarkers in GBS. Direct ROC comparisons confirmed significantly stronger discriminatory performance of NLR and SII in MG than in GBS (both *p* < 0.001).

Overall, these results demonstrate a disease-specific divergence in biomarker performance under comparable clinical conditions.

#### 3.4.3 Inflammatory indices

When inflammatory biomarkers were evaluated collectively, a clear disease-specific pattern emerged.

In MG, hemogram-derived indices, particularly NLR and SII, demonstrated consistent associations with disease severity and clinical outcomes. These findings suggest that systemic inflammatory burden parallels clinical deterioration in MG. LDH-based parameters further supported the contribution of metabolic stress to disease severity in MG. These metabolic markers likely reflect overall physiological stress and tissue injury rather than disease-specific mechanisms.

In GBS, inflammatory indices showed weaker and less consistent relationships, with outcomes primarily determined by neurological severity. Metabolic markers, particularly post-treatment transaminases, appeared to contribute more to adverse outcomes than classical inflammatory indices. These findings may reflect treatment intensity and systemic response rather than direct pathophysiological mechanisms.

**Figure 1:**
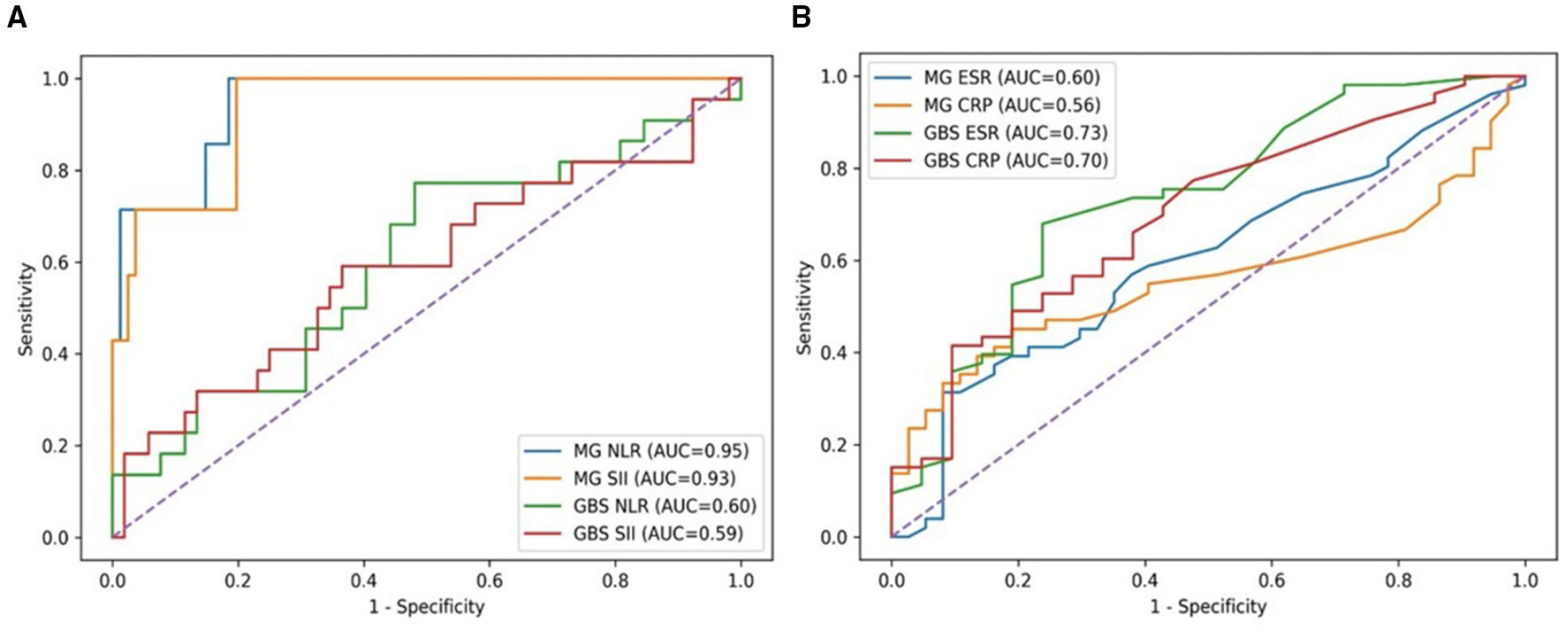
Receiver operating characteristic (ROC) analysis of inflammatory biomarkers for disease severity and prognosis in MG and GBS. **(A)** ROC curves for hemogram-derived inflammatory indices (NLR and SII). **(B)** ROC curves for classical inflammatory markers (CRP and ESR). ROC curves illustrate the discriminatory performance of biomarkers for predicting poor clinical outcome in myasthenia gravis (MGFA ≥ 4) and Guillain–Barré syndrome (GBS-DS ≥ 3). Comparative analyses between MG and GBS were performed using the DeLong test to evaluate differences in AUC values. In MG, inflammatory biomarkers demonstrated higher discriminatory performance, whereas in GBS, biomarker performance remained limited. These findings indicate that systemic inflammatory markers may reflect disease burden in MG, while clinical severity remains the dominant determinant in GBS. AUC values should be interpreted cautiously in the context of sample size and cohort characteristics.

Receiver operating characteristic analyses (Figure 1) reinforced these findings, demonstrating markedly stronger discriminatory performance of NLR and SII for poor outcome in MG compared with GBS, whereas biomarker-based discrimination in GBS remained limited. These results support a disease-specific divergence in biomarker performance under comparable neurocritical conditions. Detailed correlation coefficients are provided in Supplementary Table 2.

Multivariable models further supported these findings, with clinical severity remaining the dominant determinant of outcomes across both conditions. In MG, after adjustment for age, sex, and treatment modality, NLR and SII remained associated with higher MGFA class and increased QMG scores (NLR: MGFA (OR, 95% CI): 1.45 (1.12–1.88), QMG (β, p) +0.62; SII: MGFA (OR, 95% CI):1.31 (1.08–1.59), QMG (β, p) +0.51), whereas ESR and CRP did not retain independent significance. These associations should be interpreted within the context of disease severity rather than as independent determinants. In MV-focused models, baseline clinical severity (admission QMG) remained the dominant predictor. In GBS, none of the evaluated inflammatory biomarkers demonstrated independent associations with prognosis after adjustment, with neurological severity measures remaining the primary determinants of outcome.

## 4 Discussion

In this comparative cohort of hospitalized patients with MG and GBS requiring intensive immunotherapy, mechanical ventilation emerged as the dominant determinant of in-hospital burden. Consistent with prior literature in both disorders (4, 5, 8, 9, 12, 18–24), MV was strongly associated with prolonged length of stay. Rather than indicating a shared biomarker profile across these diseases, our findings suggest that biomarker performance diverges substantially despite a broadly comparable neurocritical care context.

Length of hospital stay paralleled these patterns. In MG, MV and LOS were associated not only with established clinical severity measures but also with systemic inflammatory and metabolic markers, particularly NLR, SII, and the LDH/albumin ratio (2–5). These associations suggest that systemic inflammatory burden and metabolic stress accompany disease severity in MG, rather than functioning as independent causal determinants of outcome. While LDH-based indices have previously been associated with adverse outcomes in systemic inflammatory conditions such as pneumonia and malignancy (11, 25), direct evidence supporting LDH as a routine prognostic biomarker in MG remains limited. Older biochemical studies in MG reported alterations in LDH activity or isoenzyme patterns, including work on serum/tissue LDH and thymus- or T-cell-related LDH profiles, but contemporary clinical studies evaluating serum LDH as a prognostic marker in MG are scarce (26, 27). Accordingly, the relevance of LDH in MG is best interpreted as reflecting a biologically plausible but underexplored axis of cellular injury and metabolic stress in an autoimmune neuromuscular disorder rather than an established disease-specific biomarker (11, 26–30). In our cohort, LDH/albumin consistently paralleled respiratory deterioration and short-term outcomes, suggesting that metabolic stress and tissue injury may represent clinically relevant components of disease burden in advanced MG. Elevated inflammatory indices and biochemical stress markers have been reported during myasthenic crisis (3–5, 31–34), and our data extend these observations by suggesting that LDH/albumin may provide complementary information alongside conventional neurological severity scales. LAR was previously associated with mortality in heart failure and other autoimmune diseases (11, 30). Because albumin also reflects systemic reserve and illness burden, the LAR may be conceptually useful as a composite marker even when LDH alone is not disease-specific (11, 29, 30).

In GBS, by contrast, MV and LOS were primarily associated with neurological disability rather than systemic inflammatory amplification. Transaminase elevations, particularly AST and ALT, showed closer relationships with adverse outcomes than classical hemogram-derived indices. Previous studies have attributed elevated transaminases in severe GBS to systemic stress, immobility, hypoxia, and treatment-related effects rather than direct immune-mediated nerve injury (9, 21–24, 35–39). Our findings are consistent with this interpretation and suggest that post-treatment ALT/AST elevations in GBS should be interpreted cautiously, particularly in cohorts where treatment intensity is strongly severity-driven. Importantly, creatine kinase (CK) was not systematically available in our cohort; therefore, we could not determine whether transaminase elevations tracked skeletal muscle injury more closely than systemic illness burden. Within our direct MG-GBS comparison, inflammatory indices added limited prognostic value beyond established neurological severity measures in GBS, reinforcing that respiratory failure in GBS largely reflects the extent of structural nerve injury and functional impairment. Notably, the proportion of axonal subtypes (AMAN/AMSAN) in our GBS cohort was higher than that reported in most Western series. This finding may reflect regional epidemiological differences, as axonal variants have been reported more frequently in certain geographic populations. Additionally, the inclusion of hospitalized patients requiring immunomodulatory therapy may have enriched the cohort for more severe phenotypes, which are known to be more frequently associated with axonal involvement. However, the relatively small sample size and single-center design limit definitive conclusions regarding subtype distribution. This distribution should be considered when interpreting the generalizability of GBS-related findings in this study.

Hemogram-derived inflammatory indices have been increasingly investigated in immune-mediated neuromuscular diseases. In MG, associations between NLR, PLR, SII, and clinical severity or crisis risk have been consistently documented (3–5, 32–34, 40). In GBS, elevated NLR and SII levels have similarly been reported, particularly in comparisons with healthy controls, with modest correlations to disability scores (6–8, 17, 37, 41). However, most prior investigations have relied on single-disease cohorts or patient-control designs, limiting evaluation of relative prognostic strength across disorders. By examining MG and GBS within the same hospitalized population, our study demonstrates that hemogram-derived indices exhibit substantially greater discriminatory performance in MG than in GBS. In MG, inflammatory burden closely paralleled clinical deterioration and poor short-term outcome; in GBS, its contribution was comparatively modest when assessed alongside validated neurological severity scales. These findings support a disease-specific, rather than uniform, interpretation of routinely available inflammatory biomarkers in neurocritical neuromuscular disorders.

The divergence observed between these disorders likely reflects fundamental differences in pathophysiology. MG is characterized by antibody-mediated disruption of neuromuscular transmission accompanied by systemic immune activation and cytokine signaling (2, 42, 43), rendering circulating inflammatory markers responsive to fluctuations in disease activity. In contrast, GBS involves acute immune-mediated structural injury to peripheral nerves (1), in which axonal damage and functional deficit primarily determine prognosis. Moreover, immunomodulatory therapies such as IVIG and plasma exchange, cornerstones of treatment in both conditions, are preferentially administered to patients with more severe presentations (44, 45). This confounding by indication should be considered when interpreting biomarker associations in observational cohorts, although diagnosis-specific parsimonious modeling was employed to reduce bias.

From a clinical perspective, these findings support a differentiated approach to biomarker interpretation. In MG, hemogram-derived indices, particularly NLR and SII, together with LDH-based parameters, may enhance contextual risk assessment for respiratory failure and prolonged hospitalization when integrated with established severity scales. However, given the limited number of MV events and the absence of external validation, these biomarkers should be considered supportive rather than standalone tools for clinical decision-making. The potential value of LDH and LAR in MG lies less in disease specificity and more in their ability to capture systemic stress, cellular injury, and physiological reserve within a clinically severe inpatient population (11, 28–30). In GBS, however, prognostic assessment should remain anchored in validated neurological severity measures, with inflammatory and metabolic markers interpreted as adjunctive indicators rather than primary determinants of outcome.

Several limitations warrant acknowledgment. The retrospective, monocentric design and predominance of IVIG treatment reflect local clinical practice patterns and may limit the generalizability of the findings and precludes causal inference. Treatment allocation was not randomized, introducing potential confounding, although diagnosis-specific multivariable models were constructed to minimize bias. Infectious triggers such as Campylobacter jejuni in GBS or recent viral infections (including COVID-19) in MG were not systematically assessed due to the retrospective design and represent an additional limitation. Biomarker measurements were confined to baseline and early hospitalization, preventing evaluation of longitudinal dynamics. Furthermore, the limited number of MV events restricts statistical precision. In addition, multiple correlation analyses were performed without formal adjustment for multiple testing; therefore, these findings should be regarded as exploratory and hypothesis-generating. Detailed sleep-related phenotyping was also unavailable. This is relevant because sleep-disordered breathing and other sleep disturbances are increasingly recognized in neuromuscular disorders, including MG, and may influence respiratory burden and quality of life; these factors were not systematically assessed in the present cohort (46, 47). Finally, although GBS subtypes were classified electrophysiologically, the cohort size limited more granular subtype-specific modeling of biomarker performance.

In conclusion, systemic inflammatory and metabolic biomarkers demonstrate disease-specific prognostic associations in hospitalized patients with MG and GBS requiring intensive immunotherapy. In MG, NLR and SII, together with LDH/albumin ratio, were associated with disease severity, respiratory failure, and short-term outcomes, underscoring the combined impact of systemic inflammatory burden and metabolic stress in advanced disease states. In GBS, short-term outcome was predominantly determined by neurological severity, with inflammatory biomarkers contributing only limited incremental value. These findings support a contextual and disease-specific framework for integrating routinely available laboratory parameters into prognostic assessment rather than applying inflammatory indices uniformly across immune-mediated neuromuscular disorders. Future prospective, multicenter studies with longitudinal biomarker profiling are warranted to validate these observations, clarify causal pathways, and determine whether dynamic changes in inflammatory and metabolic markers can further refine early risk stratification and therapeutic decision-making in these conditions. Importantly, the present findings should not be interpreted as evidence that MG and GBS are directly comparable diseases, but rather that biomarker behavior is context-dependent even within a shared neurocritical care framework.

## Data Availability

The original contributions presented in the study are included in the article/Supplementary material, further inquiries can be directed to the corresponding author.

## References

[1] Shahrizaila N Lehmann HC Kuwabara S. Guillain-barré syndrome. Lancet. (2021) 397:1214–28. doi: 10.1016/S0140-6736(21)00517-1 33647239

[2] Gilhus NE Verschuuren JJ. Myasthenia gravis: subgroup classification and therapeutic strategies. Lancet Neurol. (2015) 14:1023–36. doi: 10.1016/S1474-4422(15)00145-3 26376969

[3] Duan Z Jia A Cui W. Feng, J. Correlation between neutrophil-to-lymphocyte ratio and severity of myasthenia gravis in adults: a retrospective study. J Clin Neurosci. (2022) 106:117–21. doi: 10.1016/j.jocn.2022.10.017 36279714

[4] Yang DH Qian MZ Wei MM Li J Yu MM Lu XM Zhang X. The correlation of neutrophil-to-lymphocyte ratio with the presence and activity of myasthenia gravis. Oncotarget. (2017) 8:76099–107. doi: 10.18632/oncotarget.18546 29100295 PMC5652689

[5] Huang X Xu M Wang Y Zhang Z Li F Chen X . The systemic inflammation markers as possible indices for predicting respiratory failure and outcome in patients with myasthenia gravis. Ann Clin Transl Neurol. (2023) 10:98–110. doi: 10.1002/acn3.51706 36453129 PMC9852395

[6] Cabanillas-Lazo M Quispe-Vicuna C Cruzalegui-Bazan C Pascual-Guevara M Mori-Quispe N Alva-Diaz C. The neutrophil-to-lymphocyte ratio as a prognostic biomarker in Guillain-Barre syndrome: a systematic review with me-ta-analysis. Front Neurol. (2023) 14:1153690. doi: 10.3389/fneur.2023.1153690 37333004 PMC10272825

[7] Liu T Gao J Liu M. The clinical significance of systemic immune-inflammation index and platelet/neutrophil to lymphocyte ratio in Guillain-Barré syndrome. Clin Neurol Neurosurg. (2023) 235:108015. doi: 10.1016/j.clineuro.2023.108015 37898029

[8] Xu L Gao TX Chang SH Jiang SM Zhang LJ Yang L. Role of lymphocyte-related immune-inflammatory biomarkers in detecting early progression of Guillain-Barré syndrome. J Clin Neurosci. (2022) 105:31–6. doi: 10.1016/j.jocn.2022.08.017 36063751

[9] Orlikowski D Prigent H Sharshar T Lofaso F Raphael JC. Respiratory dysfunction in Guillain-Barré syndrome. Neurocrit Care. (2004) 1:415–22. doi: 10.1385/NCC:1:4:415 16174943

[10] Chowdhury CS Wareham E Xu J Kumar S Kofron M Lakshmikanthan S . Rap1b-loss increases neutrophil lactate dehydrogenase activity to enhance neutrophil migration and acute inflammation *in vivo*. Front Immunol. (2022) 13:1061544. doi: 10.3389/fimmu.2022.1061544 36505495 PMC9733537

[11] Meng T Ding W Lv D Wang C Xu Y. Lactate dehydrogenase to albumin ratio (LAR) is a novel predictor of fatal outcome in patients with SFTS: an observational study. Front Public Health. (2024) 12:1459712. doi: 10.3389/fpubh.2024.1459712 39741938 PMC11685223

[12] Barohn RJ McINTIRE DONALD Herbelin L Wolfe GI Nations S Bryan WW. Reliability testing of the quantitative myasthenia gravis score. Ann N Y Acad Sci. (1998) 841:769–72. doi: 10.1111/j.1749-6632.1998.tb11015.x 9668327

[13] Luo Y Dong X Peng Y Cui B Yan C Jin W . Evaluation of outcome measures for myasthenia gravis subgroups. J Clin Neurosci. (2021) 91:270–5. doi: 10.1016/j.jocn.2021.07.020 34373039

[14] Ruzhansky K Li Y Wolfe GI Muppidi S Guptill JT Hehir MK . Standardization of myasthenia gravis outcome measures in clinical practice: a report of the MGFA task force. Muscle Nerve. (2025) 72:56–65. doi: 10.1002/mus.28417 40260547

[15] Walgaard C Lingsma HF Ruts L van Doorn PA Steyerberg EW Jacobs BC. Early recognition of poor prognosis in Guillain-Barre syndrome. Neurology. (2011) 76:968–75. doi: 10.1212/WNL.0b013e3182104407 21403108 PMC3059137

[16] Kleyweg RP Van Der Meché FG Schmitz PI. Interobserver agreement in the assessment of muscle strength and functional abilities in Guillain-Barré syndrome. Muscle Nerve. (1991) 14:1103–9. doi: 10.1002/mus.880141111 1745285

[17] Sun S Wen Y Li S Huang Z Zhu J Li Y. Neutrophil-to-lymphocyte ratio is a risk indicator of Guillain-Barré syndrome and is associated with severity and short-term prognosis. Heliyon. (2023) 9:e14321. doi: 10.1016/j.heliyon.2023.e14321 36967912 PMC10036506

[18] Thomas CE Mayer SA Gungor Y Swarup R Webster EA Chang I . Myasthenic crisis: clinical features, mortality, complications, and risk factors for prolonged intubation. Neurology. (1997) 48:1253–60. doi: 10.1212/WNL.48.5.1253 9153452

[19] Alshekhlee A Miles JD Katirji B Preston DC Kaminski HJ. Incidence and mortality rates of myasthenia gravis and myasthenic crisis in US hospitals. Neurology. (2009) 72:1548–54. doi: 10.1212/WNL.0b013e3181a41211 19414721

[20] Wendell LC Levine JM. Myasthenic crisis. Neurohospitalist. (2011) 1:16–22. doi: 10.1177/1941875210382918 23983833 PMC3726100

[21] Dhar R Stitt L Hahn AF. The morbidity and outcome of patients with Guillain–Barré syndrome admitted to the intensive care unit. J Neurol Sci. (2008) 264:121–8. doi: 10.1016/j.jns.2007.08.005 17881005

[22] Van Koningsveld R Steyerberg EW Hughes RA Swan AV Van Doorn PA Jacobs BC . Clinical prognostic scoring sys-tem for Guillain–Barré syndrome. Lancet Neurol. (2007) 6:589–94. doi: 10.1016/S1474-4422(07)70130-8 17537676

[23] Walgaard C Lingsma HF Ruts L Drenthen J Steyerberg EW Jacobs BC . Prediction of respiratory insufficiency in Guillain–Barré syndrome. Ann Neurol. (2010) 67:781–7. doi: 10.1002/ana.21976 20517939

[24] Lawn ND Fletcher DD Henderson RD Wolter TD Wijdicks EF. Anticipating mechanical ventilation in Guillain–Barré syndrome. Arch Neurol. (2001) 58:893–8. doi: 10.1001/archneur.58.6.893 11405803

[25] Chai D Yang T Zhang L Hui Y Feng J Wang W. Prognostic value of the lactate dehydrogenase to albumin ratio in cancer patients. Front Nutr. (2025) 12:1610487. doi: 10.3389/fnut.2025.1610487 40693202 PMC12277156

[26] Szathmáry I Szobor A Selmeci L Pósch E. Study of lactic dehydrogenase activity and isoenzyme pattern in myasthenia gravis. Eur Neurol. (1971) 5:245–55. doi: 10.1159/000114076 5126568

[27] Szathmáry I Selmeci L Pósch E Szobor A Molnár J. Myasthenia gravis: long-term prognostic value of thymus lactate dehydrogenase isoenzyme pattern of hyperplastic thymus and thymoma. J Neurol Neurosurg Psychiatry. (1985) 48:757–61. doi: 10.1136/jnnp.48.8.757 4031927 PMC1028446

[28] Komac A Gokcen N Yazici A Cefle A. The role of lactate dehydrogenase-to-albumin ratio in clinical evaluation of adult-onset Still's disease. Int J Clin Pract. (2021) 75:e14615. doi: 10.1111/ijcp.14615 34235806

[29] Ekin A Misirci S Öztop H Hacimustafaoglu AS Coşkun BN Yagiz B . Does the LDH/albumin ratio bring novelty? a comparative analysis with inflammatory indices and combined models in adult-onset still's disease Diagnostics. (2024) 14:2780. doi: 10.3390/diagnostics14242780 39767141 PMC11674256

[30] Xia X Tan S Zeng R Ouyang C Huang X. Lactate dehydrogenase to albumin ratio is associated with in-hospital mortality in patients with acute heart failure: Data from the MIMIC-III database. Open Med (Wars). (2024) 19:20240901. doi: 10.1515/med-2024-0901 38584822 PMC10996934

[31] Dalakas MC. Immunotherapy in myasthenia gravis in the era of biologics. Nat Rev Neurol. (2019) 15:113–24. doi: 10.1038/s41582-018-0110-z 30573759

[32] Hsu CW Chen NC Huang WC Lin HC Tsai WC Huang CC . Hemogram parameters can predict in-hospital mortality of patients with Myasthenic crisis. BMC Neurol. (2021) 21:388. doi: 10.1186/s12883-021-02412-4 34615473 PMC8493047

[33] Chen T Chen H Wen Y Huang Y Lin Z Liang Q . The value of combined detection of systemic inflammation response index and prognostic nutritional index in predicting short-term prognosis of myasthenia gravis. J Inflamm Res. (2025) 18:13319–33. doi: 10.2147/JIR.S546111 41040990 PMC12484099

[34] Wang Y Huan X Zhu X Song J Yan C Yang L . Independent risk factors for in-hospital outcome of myasthenic crisis: a prospective cohort study. Ther Adv Neurol Disord. (2024) 17:17562864241226745. doi: 10.1177/17562864241226745 38344193 PMC10858673

[35] Wen P Wang L Liu H Gong L Ji H Wu H . Risk factors for the severity of Guillain-Barré syndrome and predictors of short-term prognosis of severe Guillain-Barré syndrome. Sci Rep. (2021) 11:11578. doi: 10.1038/s41598-021-91132-3 34079013 PMC8172857

[36] Yamagishi Y Suzuki H Sonoo M Kuwabara S Yokota T Nomura K . Markers for Guillain-Barré syndrome with poor prognosis: a multi-center study. J Peripher Nerv Syst. (2017) 22:433–9. doi: 10.1111/jns.12234 28833828

[37] Chen L Yang H Wu H Zhu Z Shi Z. Clinical and electrophysiological characteristics and blood markers for short-term prognosis prediction in severe Guillain-Barré syndrome: a retrospective cohort study. Mult Scler Relat Disord. (2025) 100:106532. doi: 10.1016/j.msard.2025.106532 40413968

[38] Oomes PG Van Der Meché FGA Kleyweg RP. Liver function disturbances in Guillain-Barré syndrome: a prospective longitudinal study in 100 patients. Neurology. (1996) 46:96–100. doi: 10.1212/WNL.46.1.96 8559429

[39] Zhang Y Zhao Y Wang Y. Prognostic factors of Guillain-Barré syndrome: a 111-case retrospective review. Chin Neurosurg J. (2018) 4:14. doi: 10.1186/s41016-018-0122-y 32922875 PMC7398209

[40] Bhandage AK Huang YF Punga T Punga AR. On the road to blood biomarkers in myasthenia gravis (MG): beyond clinical scales. J Neuromuscul Dis. (2025) 25:22143602251348753. doi: 10.1177/22143602251348753 40560146 PMC13437908

[41] Ethemoglu O Calik M. Effect of serum inflammatory markers on the prognosis of adult and pediatric patients with Guillain–Barré syndrome. Neuropsychiatr Dis Treat. (2018) 14:1255–60. doi: 10.2147/NDT.S162896 29805261 PMC5960237

[42] Berrih-Aknin S. Myasthenia Gravis: paradox versus paradigm in autoimmunity. J Autoimmun. (2014) 52:1–28. doi: 10.1016/j.jaut.2014.05.001 24934596

[43] Zhong H Huan X Zhao R Su M Yan C Song J . Peripheral immune landscape for hypercytokinemia in myasthenic crisis utilizing single-cell transcriptomics. J Transl Med. (2023) 21:564. doi: 10.1186/s12967-023-04421-y 37620910 PMC10464341

[44] Nandeesha SS Kasagga A Hawrami C Ricci E Hailu KT Salib K . Treatment efficacy of plasmapheresis versus intravenous immunoglobulin in Guillain-Barré syndrome management: a systematic review. Cureus. (2024) 16:e57066. doi: 10.7759/cureus.57066 38681292 PMC11052558

[45] Wang Y Huan X Jiao K Jiang Q Goh LY Shi J . Plasma exchange versus intravenous immunoglobulin in AChR sub-type myasthenic crisis: a prospective cohort study. Clin Immunol. (2022) 241:109058. doi: 10.1016/j.clim.2022.109058 35690385

[46] Cenacchi G Valentina P Marina F Elena P Corrado A. Comparison of muscle ultrastructure in myasthenia gravis with anti-MuSK and anti-AChR antibodies. J Neurol. (2011) 258:746–52. doi: 10.1007/s00415-010-5823-x 21088848

[47] Angelini CI Ansevin C Siciliano G. The role of sleep in neuromuscular disorders. Front Neurol. (2023) 14:1195302. doi: 10.3389/fneur.2023.1195302 37456652 PMC10339827

