# Supplementary material for: Disease-specific divergence of inflammatory and metabolic biomarkers in neurocritical neuromuscular disorders
Source: Front Neurol. 2026 May 13;17:1820825. doi: 10.3389/fneur.2026.1820825 (PMC13212208; doi:10.3389/fneur.2026.1820825)
Supplement: Supplementary file 1 [file Table_1.docx]

Supplementary Material

**Supplementary Table 1**. Baseline demographic, clinical, and laboratory characteristics of patients with MG and GBS.

| **Variable** | **MG**  **n = 88** | **GBS**  **n = 74** | ***p* value** | **Variable** | **MG**  **n = 88** | **GBS**  **n = 74** | ***p* value** |
| --- | --- | --- | --- | --- | --- | --- | --- |
| **Demographic and clinical characteristics** |  |  |  | **Inflammatory indices** |  |  |  |
| Male sex, % | 54.5 | 56.8 | 0.451 | SII | 1410 ± 1915 | 1341 ± 1490 | 0.800 |
| Age, years (mean ± SD) | 62.44 ± 16.4 | 57.26 ± 16.5 | **0.048** | SIRI | 357 ± 666 | 328 ± 351 | 0.737 |
| Hypertension, % | 50.0 | 44.6 | 0.530 | ESR, mm/h | 21.9 ± 18.0 | 29.4 ± 21.0 | **0.020** |
| Diabetes mellitus, % | 23.9 | 18.9 | 0.566 | CRP, mg/dL | 1.29 ± 3.8 | 1.68 ± 2.6 | 0.470 |
| Other comorbidities, % | 42.0 | 42.3 | 1.000 | **Biochemical parameters** |  |  |  |
| Mechanical ventilation, % | 9.1 | 12.2 | 0.610 | Urea, mg/dL | 37.5 ± 16.8 | 39.4 ± 17.9 | 0.486 |
| Length of hospital stay, days (mean ± SD) | 11.24 ± 15.2 | 14.20 ± 8.7 | 0.137 | Creatinine, mg/dL | 0.82 ± 0.22 | 0.91 ± 0.91 | 0.378 |
| SIADH, % (n) | 3.4 (3) | 10.8 (8) | 0.114 | Total protein, g/dL | 6.96 ± 0.8 | 7.24 ± 0.8 | **0.030** |
| **Treatment modality, %** |  |  |  | Albumin, g/dL | 3.8 ± 0.5 | 3.8 ± 0.5 | 0.618 |
| IVIG | 89.8 | 77.0 | **0.041** | Admission sodium, mmol/L | 139 ± 3.6 | 137 ± 4.0 | **<0.001** |
| Plasma exchange (PE) | 4.5 | 16.2 |  | Admission potassium, mmol/L | 4.38 ± 0.5 | 4.42 ± 0.56 | 0.639 |
| IVIG + PE | 5.7 | 6.8 |  | Admission chloride, mmol/L | 104 ± 4 | 102 ± 5 | **0.010** |
| **Hematological parameters** |  |  |  | Post-treatment sodium, mmol/L | 138 ± 3.3 | 135 ± 5.3 | **<0.001** |
| WBC (×10³/µL) | 8.63 ± 4.4 | 9.54 ± 3.9 | 0.167 | Post-treatment potassium, mmol/L | 4.1 ± 0.5 | 4.1 ± 0.5 | 0.728 |
| Hemoglobin, g/dL | 12.8 ± 2.0 | 13.5 ± 1.9 | **0.019** | Post-treatment chloride, mmol/L | 103 ± 3.4 | 100 ± 5.2 | **0.002** |
| Platelet count (×10³/µL) | 251.5 ± 74 | 280.4 ± 101 | 0.038 | Total bilirubin, mg/dL | 0.67 ± 0.34 | 0.64 ± 0.39 | 0.608 |
| Neutrophils, % | 67.7 ± 15.2 | 67.8 ± 13.5 | 0.986 | Direct bilirubin, mg/dL | 0.19 ± 0.11 | 0.17 ± 0.11 | 0.307 |
| Lymphocytes, % | 22.8 ± 12.0 | 21.7 ± 10.0 | 0.539 | GGT, U/L | 29 ± 21 | 50 ± 70 | 0.023 |
| Monocytes, % | 7.8 ± 3.6 | 7.9 ± 3.3 | 0.851 | LDH (admission), U/L | 289 ± 156 | 321 ± 130 | 0.538 |
| MCV, fL | 86.9 ± 6.9 | 85.0 ± 7.1 | 0.082 | LDH (1st week), U/L | 277 ± 190 | 258 ± 107 | 0.524 |
| Hematocrit, % | 38.4 ± 5.6 | 40.0 ± 5.0 | 0.062 | LDH/albumin (admission) | 80.18 ± 50.2 | 84.9 ± 35.8 | 0.538 |
| **Inflammatory indices** |  |  |  | LDH/albumin (1st week) | 76.76 ± 59.2 | 70.6 ± 38.8 | 0.528 |
| NLR | 6.3 ± 11.1 | 4.5 ± 3.8 | 0.119 | ALT (admission), U/L | 19.68 ± 10.9 | 29.04 ± 23.7 | **0.001** |
| PLR | 2.1 ± 1.6 | 1.8 ± 1.0 | 0.203 | AST (admission), U/L | 23.11 ± 8.5 | 31.88 ± 14.6 | **<0.001** |
| MLR | 0.45 ± 0.45 | 0.43 ± 0.26 | 0.784 | ALT (after treatment), U/L | 32.09 ± 33.8 | 56.24 ± 68.0 | **0.004** |
| LMR | 3.3 ± 1.9 | 3.5 ± 4.3 | 0.621 | AST (after treatment), U/L | 37.33 ± 37.8 | 46.64 ± 37.3 | 0.118 |

Supplementary Table 1. Extended baseline demographic, clinical, and laboratory characteristics of patients with MG and GBS. Values are presented as mean ± standard deviation or percentage, as appropriate. Comparisons between groups were performed using the independent-samples t test or Mann–Whitney U test for continuous variables, and the χ² test or Fisher’s exact test for categorical variables. This table provides the full set of baseline laboratory parameters to ensure transparency and completeness of the dataset. Variables not included in the main Table 1 were excluded from the primary presentation to improve clarity and focus on clinically relevant findings. Observed differences should be interpreted in the context of disease-specific characteristics and overall clinical status. SIADH: syndrome of inappropriate antidiuretic hormone secretion; ESR: erythrocyte sedimentation rate; CRP: C-reactive protein; GGT: gamma-glutamyl transferase.
