# Supplementary material for: Disease-specific divergence of inflammatory and metabolic biomarkers in neurocritical neuromuscular disorders
Source: Front Neurol. 2026 May 13;17:1820825. doi: 10.3389/fneur.2026.1820825 (PMC13212208; doi:10.3389/fneur.2026.1820825)
Supplement: Supplementary file 2 [file Table_2.docx]

Supplementary Material

**Supplementary Table 2**

**Table 2 A**. Correlations between disease severity, prognosis, and inflammatory biomarkers in MG.

| **Variable** | **LOS** | **MV** | **MGFA** | **Admission QMG** | **Discharge QMG** | **QMG Difference** | **6th Month QMG** | **1st Year QMG** |
| --- | --- | --- | --- | --- | --- | --- | --- | --- |
| **SII** | *p*=0.005 r=0.299 | *p*<0.001 r=**0.617** | *p*<0.001 r=**0.548** | *p*<0.001 r=0.498 | *p*<0.001 r=0.473 | *p*=0.002 r=0.322 | *p*=0.99 r=0.001 | *p*=0.65 r=−0.056 |
| **SIRI** | *p*=0.074 r=0.192 | *p*<0.001 r=**0.550** | *p*<0.001 r=0.494 | *p*<0.001 r=0.446 | *p*<0.001 r=0.386 | *p*=0.001 r=0.342 | *p*=0.06 r=0.222 | *p*=0.44 r=0.097 |
| **NLR** | *p*=0.002 r=0.320 | *p*<0.001 r=**0.615** | *p*<0.001 r=**0.546** | *p*<0.001 r=0.449 | *p*<0.001 r=0.415 | *p*<0.001 r=0.407 | *p*=0.56 r=0.071 | *p*=0.89 r=−0.016 |
| **MLR** | *p*=0.065 r=0.197 | *p*<0.001 r=**0.502** | *p*<0.001 r=0.399 | *p*<0.001 r=0.370 | *p*=0.023 r=0.243 | *p*<0.001 r=0.392 | *p*=0.15 r=0.173 | *p*=0.20 r=0.161 |
| **LMR** | *p*=0.49 r=−0.074 | *p*=0.13 r=−0.163 | *p*=0.287 r=−0.115 | *p*=0.14 r=−0.159 | *p*=0.49 r=−0.074 | *p*=0.050 r=−0.210 | *p*=0.07 r=−0.046 | *p*=0.65 r=−0.058 |
| **PLR** | *p*=0.012 r=0.265 | *p*<0.001 r=0.431 | *p*=0.002 r=0.322 | *p*=0.009 r=0.277 | *p*=0.042 r=0.217 | *p*=0.022 r=0.243 | *p*=0.33 r=−0.119 | *p*=0.59 r=−0.067 |
| **CRP** | *p*=0.22 r=0.15 | *p*=0.0004 r=0.369 | *p*=0.0049 r=0.299 | ns | ns | ns | ns | ns |
| **ESR** | *p*=0.0068 r=0.286 | *p*=0.051 r=0.209 | ns | ns | *p*=0.034 r=0.226 | ns | ns | ns |

**Table 2 B**. Correlations between disease severity, prognosis, and inflammatory biomarkers in GBS.

| **Variable** | **LOS** | **MV** | **GBS-DS** | **Prognosis (Poor)** | **Sev mEGOS** | **Admission MRC** | **Discharge MRC** | **6th Month MRC** | **1st Year MRC** |
| --- | --- | --- | --- | --- | --- | --- | --- | --- | --- |
| **SII** | *p*<0.001 r=0.446 | *p*=0.096 r=0.195 | *p*=0.017 r=0.277 | *p*=0.097 r=0.194 | *p*=0.024 r=0.262 | *p*=0.27 r=−0.129 | *p*=0.034 r=−0.247 | *p*=0.097 r=−0.240 | *p*=0.71 r=0.056 |
| **SIRI** | *p*=0.026 r=0.256 | *p*=0.08 r=0.205 | *p*=0.11 r=0.186 | *p*=0.48 r=0.083 | *p*=0.23 r=0.139 | *p*=0.026 r=−0.258 | *p*=0.24r =−0.137 | *p*=0.73 r=−0.050 | *p*=0.46 r=0.111 |
| **NLR** | *p*<0.001 r=0.444 | *p*=0.030 r=0.253 | *p*=0.007 r=0.310 | *p*=0.041 r=0.238 | *p*=0.013 r=0.287 | *p*=0.22 r=−0.143 | *p*=0.017 r=−0.277 | *p*=0.15 r=−0.207 | *p*=0.59 r=0.080 |
| **MLR** | *p*=0.051 r=0.228 | *p*=0.35 r=0.109 | *p*=0.188 r=0.155 | *p*=0.51 r=0.078 | *p*=0.20 r=0.150 | *p*=0.005 r=−0.324 | *p*=0.22 r=−0.143 | *p*=0.97 r=0.005 | *p*=0.20 r=0.190 |
| **LMR** | *p*=0.071 r=−0.211 | *p*=0.46 r=−0.086 | *p*=0.36 r=−0.107 | *p*=0.38 r=−0.103 | *p*=0.199 r=−0.151 | *p*=0.149 r=0.170 | *p*=0.198 r=0.151 | *p*=0.63 r=0.070 | *p*=0.40 r=−0.126 |
| **PLR** | *p*=0.001 r=0.377 | *p*=0.93 r=0.010 | *p*=0.099 r=0.193 | *p*=0.24 r=0.136 | *p*=0.049 r=0.230 | *p*=0.132 r=−0.177 | *p*=0.070 r=−0.212 | *p*=0.17 r=−0.197 | *p*=0.78 r=0.042 |
| **CRP** | ns | ns | ns | ns | ns | ns | ns | ns | ns |
| **ESR** | ns | ns | ns | ns | ns | ns | ns | ns | ns |

A: Myasthenia gravis. Correlation analyses were performed using Pearson or Spearman correlation tests, as appropriate. Strength of correlation was defined as |r| ≥ 0.50 (strong), 0.30–0.49 (moderate), and 0.10–0.29 (weak). Correlation analyses are presented to provide a comprehensive overview of associations between inflammatory biomarkers and clinical parameters. Given the large number of comparisons, these analyses should be interpreted as exploratory and hypothesis-generating. Observed associations do not imply causality and should be interpreted in the context of overall disease severity.MGFA: Myasthenia Gravis Foundation of America classification; QMG: Quantitative Myasthenia Gravis score; LOS: length of hospital stay; MV: mechanical ventilation. NLR: neutrophil-to-lymphocyte ratio; PLR: platelet-to-lymphocyte ratio; MLR: monocyte-to-lymphocyte ratio; LMR: lymphocyte-to-monocyte ratio; SII: systemic immune-inflammation index; SIRI: systemic inflammation response index; ESR: erythrocyte sedimentation rate; CRP: C-reactive protein; ns: not significant.

B: Guillain–Barré syndrome. Correlation analyses were performed using Pearson or Spearman correlation tests, as appropriate. Prognosis was categorized as good (GBS-DS 0–2) or poor (GBS-DS 3–6). Strength of correlation was defined as |r| ≥ 0.50 (strong), 0.30–0.49 (moderate), and 0.10–0.29 (weak). These analyses demonstrate that correlations between inflammatory biomarkers and clinical outcomes are generally weaker in GBS compared with MG. Clinical outcomes in GBS are predominantly associated with neurological severity measures rather than systemic inflammatory markers. As in panel A, results should be interpreted as exploratory and in the context of multiple comparisons. GBS-DS: Guillain–Barré Syndrome Disability Score; Sev mEGOS: the most severe modified Erasmus GBS Outcome Score; MRC: Medical Research Council sum score; LOS: length of hospital stay; CRP: C-reactive protein; ESR: erythrocyte sedimentation rate; other abbreviations as in Table 1; ns: not significant.
